# Inferring Insulin Secretion Rate from Sparse Patient Glucose and Insulin Measures

**DOI:** 10.3389/fphys.2022.893862

**Published:** 2022-08-03

**Authors:** Rammah M. Abohtyra, Christine L. Chan, David J. Albers, Bruce J. Gluckman

**Affiliations:** ^1^ Center for Neural Engineering, The Pennsylvania State University, University Park, PA, United States; ^2^ Department of Engineering Science and Mechanics, The Pennsylvania State University, University Park, PA, United States; ^3^ Section of Pediatric Endocrinology, University of Colorado School of Medicine, Aurora, CO, United States; ^4^ Department of Bioengineering, University of Colorado School of Medicine, Aurora, CO, United States; ^5^ Department of Neurosurgery, College of Medicine, The Pennsylvania State University, University Park, PA, United States; ^6^ Department of Biomedical Engineering, The Pennsylvania State University, University Park, PA, United States

**Keywords:** estimation algorithm, ISR function, compartment models, insulin and C-peptide, OGTT, and CSR/ISR molar ratio

## Abstract

The insulin secretion rate (ISR) contains information that can provide a personal, quantitative understanding of endocrine function. If the ISR can be reliably inferred from measurements, it could be used for understanding and clinically diagnosing problems with the glucose regulation system.

**Objective**: This study aims to develop a model-based method for inferring a parametrization of the ISR and related physiological information among people with different glycemic conditions in a robust manner. The developed algorithm is applicable for both dense or sparsely sampled plasma glucose/insulin measurements, where sparseness is defined in terms of sampling time with respect to the fastest time scale of the dynamics.

**Methods:** An algorithm for parametrizing and validating a functional form of the ISR for different compartmental models with unknown but estimable ISR function and absorption/decay rates describing the dynamics of insulin accumulation was developed. The method and modeling applies equally to c-peptide secretion rate (CSR) when c-peptide is measured. Accuracy of fit is reliant on reconstruction error of the measured trajectories, and when c-peptide is measured the relationship between CSR and ISR. The algorithm was applied to data from 17 subjects with normal glucose regulatory systems and 9 subjects with cystic fibrosis related diabetes (CFRD) in which glucose, insulin and c-peptide were measured in course of oral glucose tolerance tests (OGTT).

**Results:** This model-based algorithm inferred parametrization of the ISR and CSR functional with relatively low reconstruction error for 12 of 17 control and 7 of 9 CFRD subjects. We demonstrate that when there are suspect measurements points, the validity of excluding them may be interrogated with this method.

**Significance:** A new estimation method is available to infer the ISR and CSR functional profile along with plasma insulin and c-peptide absorption rates from sparse measurements of insulin, c-peptide, and plasma glucose concentrations. We propose a method to interrogate and exclude potentially erroneous OGTT measurement points based on reconstruction errors.

## 1 Introduction

Insulin is the essential hormone that regulates cellular energy supply and the intracellular transport of glucose into muscle and adipose tissues (Wilcox, 2005). The endogenous insulin secretion rate (ISR) quantifies the amount of insulin the body is able to produce as a function of glucose concentration in the blood, providing important information for understanding how an individual’s endocrine system is able to use insulin to regulate glucose regulation. The primary physiological stimulation for insulin secretion from beta-cell is elevated blood glucose levels following nutrition intake and glucose bolus (Ahrén and Pacini, 2004).

The objective of this work is to provide a methodology to infer the *functional form* of the ISR from insulin and glucose measurements at a personalized level that is robust to outliers.

Our motivation for this objective is threefold: First, from a clinical diagnostic standpoint, the ISR is a measure of the input/output function of a segment of the glucose regulation system - the pancreatic beta cells - and therefore would allow monitoring of their health or disease progression; second, accurate parametrization of the functional performance of the beta cells will allow more accurate modeling of the glucose regulation system and therefore understanding of normal and abnormal glucose regulation; and third, personalized ISR estimation combined with better modeling will allow for better interpretation of aberrant dynamics observed in standard glucose monitoring protocols.

In addition, glucose tolerance tests are intrusive and burdensome for subjects and have a relatively high rate of error due to outliers which limits their usefulness at a population scale. The development of an ISR estimation method that is robust to outliers, or able to identify and exclude outliers, increases the practical applicability of such tests.

**FIGURE 1:**
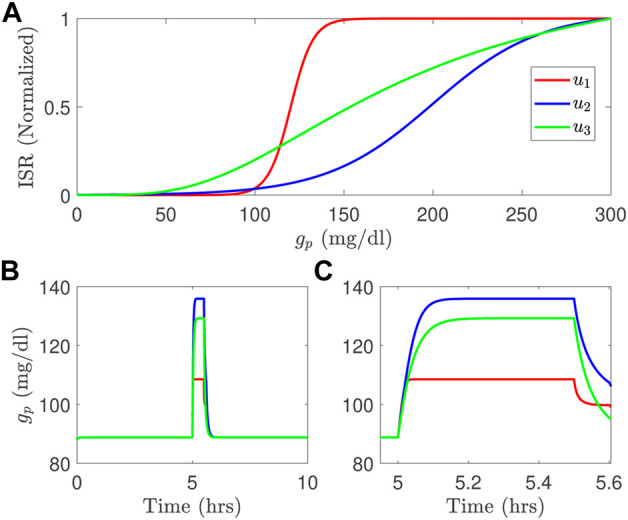
Three different ISR functions **(A)** generate blood glucose variations, in long time course **(B)** and short time course **(C)** simulated using the model developed by Topp et al. (2000) with a meal; *u*_1_ is the ISR function adopted from Liu et al. (2009); *u*_2_ is the ISR used in Tolić et al. (2000), and *u*_3_ is the ISR function used in the model of Ha et al. (2016).

Computational models of glucose regulation do already exist and have embedded in them model components for beta-cell function. But different models invoke significantly different functions for the ISR, as illustrated in the Figure 1A for the studies in (Tolić et al., 2000; Liu et al., 2009; Ha et al., 2016). These different ISR functions lead to significantly different glucose dynamics if used interchangeably within the same glucose regulation model for the same system input, as illustrated in the Figures 1B,C. Note that the functional forms change both the height and time course of the blood glucose response.

The most common methods to estimate ISR utilize plasma insulin and c-peptide concentration measurements. C-peptide (connecting peptide) is an amino acid polypeptide that is released, along with insulin, from the pancreatic beta cells when proinsulin is split into insulin and c-peptide (Steiner et al., 1967; Rigler et al., 1999), at a molar release ratio of 1:1 (ISR to c-peptide secretaoin rate CSR) (Lebowitz and Blumenthal, 1993). C-peptide is often used to distinguish insulin produced by the body from injected insulin to estimate ISR, to determine insulin resistance, and to indicate a differential diagnosis of fasting hypoglycemia with hyperinsulinism. Pancreatic beta cells release both insulin and c-peptide directly into the blood stream in the portal vein, which then passes through the liver and then combines with the rest of the circulating blood. Insulin is sensed by hypatocrytes, and signals them to start glucose uptake, and inhibit gluconeogenesis, glycogenolysis, and ketogenesis (Brundin, 1999), and at high levels to activate carcino-embryonic antigen-related cell adhesion molecule 1 (CEACAM1) to increase hypatic insulin clearance (Najjar and Perdomo, 2019). In contrast to insulin, c-peptide is primarily degraded by the kidneys (Jones and Hattersley, 2013). Insulin is degraded within 15–30 min in the bloodstream (Farris et al., 2003), while c-peptide degradation is longer (Leighton et al., 2017).

Glucose tolerance, insulin resistence, and insulin secretion in a clinical setting are generally measured with various types of glucose tolerance tests. These tests include the intravenous glucose tolerance test (IVGTT) (Bergman et al., 1981), fasting glucose assessment (Matthews et al., 1985; Pacini and Mari, 2003), and the oral glucose tolerance test (OGTT) (Ferrannini and Mari, 2004). IVGTT are less frequently performed because they are invasive and challenging to endure to the patient and expensive to achieve (Lotz et al., 2009) because of the frequent sampling protocols of the c-peptide up to every minute during an IVGTT. The more commonly used OGTT requires fasting patients to ingest a drink with a fixed amount of glucose followed by glucose measurements every 15–30 min over the subsequent two to 4 hours (Reaven et al., 1993).

Several model-based estimation methods have been developed to estimate ISR in the sense of the time course of insulin production. One approach is to estimate from insulin and c-peptide measurements (Watanabe et al., 1998; Watanabe and Bergman, 2000; Kjems et al., 2001; Venugopal et al., 2021). These multiple compartment methods treat ISR as an unknown time trajectory either without *a priori* knowledge of its secretion rate function or with different functions to describe the secretion rate. For example, in (Kjems et al., 2001), the deconvolution method is used to estimated ISR by modeling ISR with two exponential functions (biexponential model) with unknown parameters. Another approach that has both one-compartment model (Watanabe et al., 1998) and two-compartment model (Watanabe and Bergman, 2000) forms is used to estimate the time traces of ISR using a smoothed c-peptide profile generated by cubic spline interpolation. More recently, Venugopal et al. (2021) developed a method to estimate ISR using the Oral c-peptide Minimal Model (OCMM). This method describes the ISR function by two rates proportional linearly with the c-peptide and glucose concentrations. Another recent estimation approach based on OGTT measurements of insulin and c-peptide has been developed to estimate the ISR time traces using two different models, for insulin and c-peptide (Schiavon et al., 2021).

To this end, we develop a new estimation algorithm to infer the ISR from glucose/insulin measurements such as OGTT data. Using a compartmental model for the accumulation/degradation of insulin similar to (Tolić et al., 2000; Liu et al., 2009; Ha et al., 2016), this new method begins with a parametrization of the form of the ISR function that takes glucose concentration as an input and then estimates the parametrization parameters by minimizing the difference between the model output and insulin measurements. The same accumulation/degradation model and inference method can be used to independently infer the c-peptide secretion rate from glucose/c-peptide data when available.

We note that the method we derive is not reliant on the experimental protocol being an OGTT, nor that all the data are measured densely with respect to the fastest time scale of the glucose or insulin dynamics. This time is estimated in the literature to be on the order of 8–20 min for circulating glucose/insulin dynamics, and faster if one is trying to resolve the pulsitivity of insulin production. In this sense it works with sparsely sampled data. This definition is in contrast to terms in the literature that refer to OGTTs with less than 7, and as little as 3, measurement points as ‘sparse OGTTs’.

When both insulin and c-peptide are available, because the ISR and CSR functionals are independently inferred, we can use the expected 1:1 M ratio to validate the estimates and to identify data-related errors.

Our approach provides physiological insights into beta-cell secretion rates for people with different ISR health conditions. We validate the performance of the approach using OGTT clinical data for control and CFRD subjects.

## 2 Materials and Methods

The proposed algorithm uses parametric models including a single and a two-compartment model, and ISR and CSR function forms with physiological parameters. The parameters of these models and ISR/CSR functions are assumed unknown, but can be inferred from patient data, including plasma glucose, insulin, and c-peptide measurements. We test the performance of this algorithm using OGTT clinical data collected from control and CFRD subjects.

### 2.1 Human Oral Glucose Tolerance Test Data

Data used is a subset of data collected under the GlycEmic Monitoring in CF (GEM-CF, NCT02211235), a study of early glucose abnormalities in youth with cystic fibrosis. The study was approved by the Colorado Multiple Institutional Review Board (Aurora, CO), and informed consent and assent obtained. Collection details have been previously published in (Tommerdahl et al., 2021; Chan et al., 2022).

In short, inclusion criteria for participants with CFRD included a confirmed diagnosis of CFRD by newborn screen, sweat chloride testing, or genetic testing. Exclusion criteria for participants with CFRD included known Type 1 or Type 2 diabetes, use of medications affecting glucose (eg, insulin, systemic steroids) in the prior 3 months, hospitalization in the prior 6 weeks, or pregnancy. For this report, n = 9 youth with CFRD were included. N = 3 (33%) were male. CFRD individuals were an average age of 14.6 ± 3.2 years with a mean BMI of 19.0 ± 2.7 kg/m^2^ and BMI z-score of - 0.28 ± 0.53. Glucose tolerance categories by OGTT were as follows—6 CFRD patients had CFRD based on 2 h OGTT glucose > 200 mg/dl and 3 were classified as NGT. The CF cohort had an average A1C of 5.7 ± 0.2%.

Healthy controls without CFRD were identified using recruitment flyers and emails at the University of Colorado Anschutz Medical Campus. Exclusion criteria for healthy controls (HCs) included diagnoses of diabetes or prediabetes, overweight (defined as BMI ≥85th% by the Centers for Disease Control and Prevention BMI growth charts in youth), chronic disease, acute illness, or pregnancy. A total of *n* = 17 HCs were included of which n = 9 (53%) were male. HCs had an average age of 13.3 ± 3.6 years, BMI of 18.5 ± 2.9 kg/m^2^, and BMI z-score of −0.20 ± 0.68. The HCs had an average A1C of 5.3 ± 0.2%.

Subjects underwent a standard OGTT protocol, with blood drawn at times.*t*_*i*_ ∈ { − 10, 0, 20, 30, 60, 90, 120, 150, 180} min, and assayed for plasma glucose, insulin, and c-peptide concentrations.

### 2.2 Insulin and C-peptide Models

**FIGURE 2:**
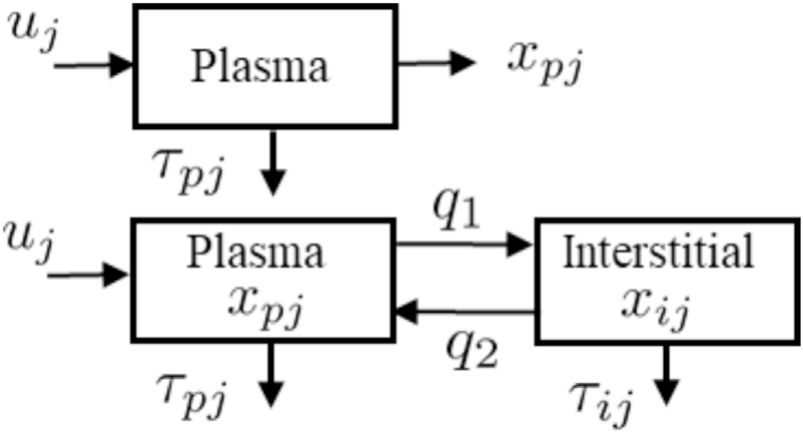
Schematic representations for the single-compartment model (Top) used to describe plasma insulin and c-peptide, and two-compartment model (Bottom) used to describe both plasma and interstitial insulin and c-peptide.

The two models, described in Figure 2, are used in the algorithm to reconstruct ISR and CSR. These models, include a single and a two-compartment model both of which use the same ISR and CSR function but with different parameters to describe the time evolution of plasma insulin and c-peptide. The single compartment model consists of a single plasma pool with a degradation time for plasma insulin and c-peptide. On the other hand, the two-compartment model tracks insulin and c-peptide concentrations in both plasma and interstitial compartments.

### 2.2.1 Single Compartment Model

The detail of the single model (Figure 2 Top) is parameterized as follows. The pancreatic beta-cell, which has a nonlinear output secretion function, is denoted by *u*_*j*_, the subscript *j* is an index that takes *I* for insulin and *Cpep* for c-peptide, and releases insulin and c-peptide using various physiological parameters. The subscript *p* denotes plasma, *τ*_*pI*_ and *τ*_*Cpep*_ denote the degradation time for the plasma insulin and c-peptide, respectively. The single compartment model is given by the equation:

$${\dot{x}}_{pj}=u_{j}\left(t\right)-x_{pj}/\tau_{pj}$$ (1)

where *x*_*pj*_ is plasma insulin or c-peptide, and *τ*_*pj*_ is the associated degradation time.

Following (Tolić et al., 2000; Liu et al., 2009), we use a sigmodal function, which is glucose dependent, for both ISR (*u*_*pI*_) and CSR (*u*_*pC*_) are given by

$$u_{j}\left(g_{p}\left(t\right)\right)=\frac{K_{m}}{1+e^{\left(\alpha \left(C_{0}-g_{p}\left(t\right)\right)\right)}}.$$ (2)

Here, *g*_*p*_(*t*) (mg/dl) is the plasma glucose concentration at a given time *t* (min), *K*_*m*_ represents a maximum production rate for insulin (*μ*U/ml/min) or c-peptide (ng/ml/min), *C*_0_ refers to a glucose mid-point (mg/dl), and *α* represents 1/width (dl/mg) of the sigmoid curve.

We combine the unknown parameters of the single compartment model in this vector Θ_*s*_:

$$\Theta_{s}={\left[\tau_{pj},K_{m},C_{0},\alpha \right]}^{T}.$$ (3)

### 2.2.2 Two Compartment Model

The two compartmental model, as shown in Figure 2 (Bottom), is comprised of two equations:

$${\dot{x}}_{pj}=u_{j}+q_{2}x_{ij}-\left(q_{1}+1/\tau_{pj}\right)x_{pj}$$ (4a)

$${\dot{x}}_{ij}=q_{1}x_{pj}-\left(q_{2}+1/\tau_{ij}\right)x_{ij}$$ (4b)

where *x*_*pj*_ and *x*_*ij*_ represent the insulin (or c-peptide) concentrations in the plasma (*p*) and interstitial (*i*) compartments; *q*_1_ and *q*_2_ represent the mass transport between these two compartments; *τ*_*pj*_ and *τ*_*ij*_ refer to the degradation time for insulin or c-peptide in the plasma and interstitial spaces. The values of *q*_1_ = 0.0473 (min^−1^) and *q*_2_ = 0.0348 (min^−1^) are adopted from the transport model of (Eaton et al., 1980). Alternatives to this model include the diffusive transport used, for example, in the ultradian model (Tolić et al., 2000). We combine the unknown parameters of the two compartment model in this vector Θ_*m*_:

$$\Theta_{m}={\left[\tau_{pj},\tau_{ij},K_{m},C_{0},\alpha \right]}^{T}$$ (5)

Finally, we provide a summary for the two models given in Eq. 1 and Eq. 4, as follows:

• The accumulation dynamics of the insulin and c-peptide use the same compartment models but with different parameters.
• *u* uses the same function for both ISR and CSR, and this function depends only on the blood glucose values.
• The function of $u\left(\bar{g}\right)$ is given by a sigmoid Eq. 2 as a function of interpolated (at 1 min) blood glucose values $\bar{g}$ , described in the next section.
• The parameters of *u* (*K*_*m*_, *C*_0_, *α*), along with degradation time (*τ*_*pj*_, *τ*_*ij*_), are unknown and estimated independently from insulin and c-peptide measurements.

### 2.2.3 Compact Form Model

It is convenient and, as we’ll show, computationally efficient to express these compartmental models in a compact state-space model form:

$$\dot{x}=A\left(\Theta \right)x+Bu\left(\Theta ,g_{p}\right)$$ (6a)

y=Cx (6b)

where *x*, *A*, *B*, *C*, and **Θ** are specific model state and parameters. For the single compartment model (2), we have *x* = *x*_*pj*_, *A* = 1/*τ*_*pj*_, *B* = 1, *C* = 1, and **Θ** = **Θ**_*s*_, which is defined in Eq. 3. For the two compartment model (4), we have $x={\left[x_{pj},x_{ij}\right]}^{T}$ ,

$$A=\left[\begin{array}{cc} -\left(q_{1}+1/\tau_{pj}\right) & q_{2}\\ q_{1} & -\left(q_{2}+1/\tau_{ij}\right) \end{array}\right]$$ (7)

*B* = [1,0]^*T*^, *C* = [1, 0], and **Θ** = **Θ**_*m*_ defined in Eq. 5.

Since we use discrete-time data, the state space model, (6), is discretized at a sampling rate of *T*_*s*_ = 0.1 min and then given by

$$x_{k+1}=\;\Phi \left(\Theta \right)x_{k}+\Gamma \left(\Theta \right)u_{k}\left(\Theta ,\bar{g}\right)$$ (8a)

$$y_{k}=\;Cx_{k}$$ (8b)

where *Φ* = *e* ^*AT*^ and $\Gamma =\int_{0}^{T}e^{ATs}dsB$ . The input *u*_*k*_ is the ISR or CSR, which is a function of both **Θ** and the interpolated glucose values $\bar{g}$ generated from cubic interpolation method.

## 3 Results

The main contribution of this paper is the development of a new estimation approach to infer the ISR from data. The uncertainty in the estimation is studied based on random initial conditions used with the proposed approach to optimize the unknown parameters.

### 3.1 The Estimation Algorithm

Our new estimation method utilizes the above state space model, (8), and is based on the nonlinear least square method (Hansen et al., 2013) to optimize parameters that provide the best fit between the model’s output (blood insulin or c-peptide) and data. The proposed algorithm uses interpolated blood glucose values as an input to the algorithm.

In practice, the time intervals of the measured blood glucose varies between 10 and 30 min, and are assumed to sufficiently cover shape of the glucose dynamics. This allows us to interpolate the glucose dynamics in order to resample the glucose values with sufficient time resolution to integrate the insulin or c-peptide dynamics, for which these measured time intervals are too long (sparse). We use cubic interpolation to resample the blood glucose values between the actual measurements to generate an interpolated glucose trajectory $\left(\bar{g}\right)$ with a time step *T* = 1 min. This input glucose trajectory is used within the ISR or CSR function $u\left(\Theta ,\bar{g}\right)$ to integrate the model forward generating a model insulin or c-peptide trajectory *y* (**Θ**, *t*). We take values from this trajectory at *t*_*k*_, which are the times of the actual insulin or c-peptide measurements, and use them in the algorithm to optimize the parameters.

**FIGURE 3:**
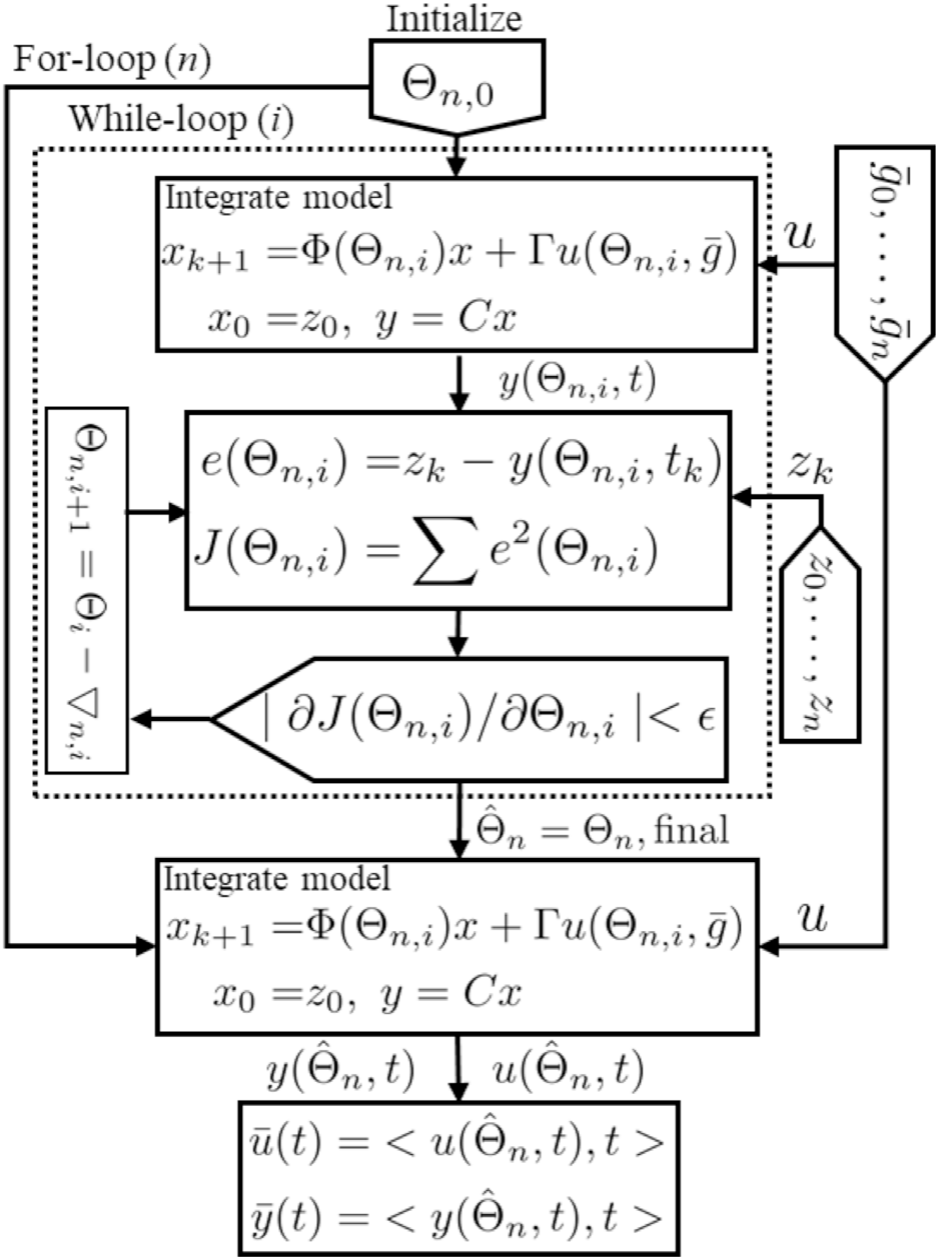
Schematic representation for the estimation algorithm.

For given measurements of glucose and blood insulin or c-peptide: *z* (1), *z* (2), …, *z*(*n*), we minimize following least squares objective function *J*(**Θ**) to obtain $\hat{\Theta }$ :

$$J\left(\Theta \right)=\overset{N}{\underset{k=0}{\sum }}{\left(z\left(k\right)-y\left(\Theta ,t_{k}\right)\right)}^{2}$$ (9)

where *y* (**Θ**, *t*_*k*_) is the model output (blood insulin or c-peptide) generated by $\bar{g}$ , and *z*(*k*) is a measured insulin or c-peptide value. Note that insulin and c-peptide are optimized independently. In Figure 3, we provide a schematic representation for our Algorithm 1. The algorithm consists of two nested loops: the outer one loops over a random set of initial conditions {**Θ**_*n*,0_}, and the inner loop is based on the Levenberg-Marquardt method where the value Θ_*n*_ is updated on the *i*th cycle by Θ_*n*,*i*+1_ = Θ_*n*,*i*_ − ∇_*n*,*i*_ where ∇_*n*,*i*_ uses the steepest descent method (Marquadt, 1963; Levenberg, 1944). We use the MATLAB function ‘lsqcurvefit’ to implement this inner loop.

### 3.1.1 Uncertainty Quantification

The method as described is a nonlinear optimization process. It is not known or proven that for this process there is either a global minimum, or only one local minimum, of the objective function (Eq. 9). Therefore there is potential sensitivity to the initial conditions (initial guess for **Θ**). To address this, and to provide uncertainty quantification for the inferred parameterization, we adopt a bootstrap method.

We therefore explore the distribution of inferred parameters $\hat{\Theta }$ from a large (1,000) randomly sampled initial conditions drawn from a range of allowed parameters defined within physiologically plausible ranges. In this analysis, *τ*_*px*_ ∈ [10, 180] min, *C*_0_ ∈ [200, 1,500] mg/L, *K*_*m*_ ∈ [1, 350] mU/l/min, and *α* ∈ [0.015, 0.045] L/mg.

For each initial parameter **Θ**_**0**_ the algorithm seeks a final parameter **Θ**_*f*_ that minimizes *J* (Eq. 9) within the boundaries. If the minimization process reaches the allowed boundaries, the result is excluded.

Each solution in **Θ**_***f***_ is used within the ISR/CSR function to simulate ISR and CSR trajectories and then generate plasma insulin and c-peptide trajectories by integrating the model, (8), forward using the interpolated glucose values as an input. Finally, we use these trajectories to compute the average and standard deviation (Mean ± SD). These steps are illustrated in Algorithm 1.

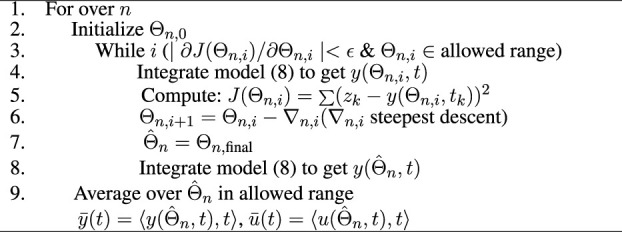

### 3.2 Computational Method Validation

We validate the inference method by applying the algorithm to model-generated data sets. We then compare the inferred ISR parametrization, and decay constant, to the model parameters used to generate the data.

Data sets were generated with the model described in Liu et al. (2009), with the published parameters unless otherwise noted. The ISR used matched the functional form in Eq. 2, with parameters *K*_*m*_ = 0.6 (mU/l/min), *C*_0_ = 1,000 (mg/L), *α* = 0.01 (L/min). Data sets were generated for each of the following insulin degradation rates *τ*_*pI*_ ∈ {10, 15, 30, 60, 90, 120} min. The model was driven with an OGGT type feeding function, glucose and insulin values sampled at discrete times *T*_*i*_ ∈ { − 10, 0, 10, 20, 30, 60, 90, 120, 150, 180} min, and 20% random noise was added.

The inferred values of *τ*_*pI*_ matched the ideal (generating) values within 1.8% (i.e., |*τ*_*pI*,*i*_ − *τ*_*pI*_|/*τ*_*pI*_ < 1.8%), and the other parameters (*K*_*m*_, *C*_0_, *α*) within a roughly average error of 1.2% of the generating parameter value.

We note that we achieved a very high level of accuracy in inference of these parameters almost independent of how sparsely the data was sampled with respect to the insulin dynamics (*τ*_*pI*_, and robust to the presence of significant (20%) added measurement noise.

### 3.3 Application to Oral Glucose Tolerance Test Data

We use this method with clinically measured OGTT data, including plasma glucose, insulin, and c-peptide measurements, to parametrize the ISR/CSR functions $\left(\hat{\Theta }\right)$ .

These measurements are taken from normal and CFRD subjects at times *t*_*i*_ = { − 10, 0, 20, 30, 60, 90, 120, 150, 180} min. The glucose values are interpolated at 1 min intervals using cubic interpolation and then used as an input for estimation, and as described $\hat{\Theta }$ is the mean parameters over minimizations of the cost function *J*(**Θ**) (Eq. 9).

Given that insulin and c-peptide are secreted in a 1:1 M ratio (Lebowitz and Blumenthal, 1993), we expect that the parametrized functionals ISR and CSR should follow a similar linear relationship. Note that in the presented units for ISR (*μ* U/ml-min) and CSR (ng/ml-min), a 1:1 M ratio corresponds to 0.056 = ng/*μ* U. We therefore also fit the relation between CSR and ISR with a linear fit to get $\tilde{CSR}\left(ISR\right)$ .

**FIGURE 4:**
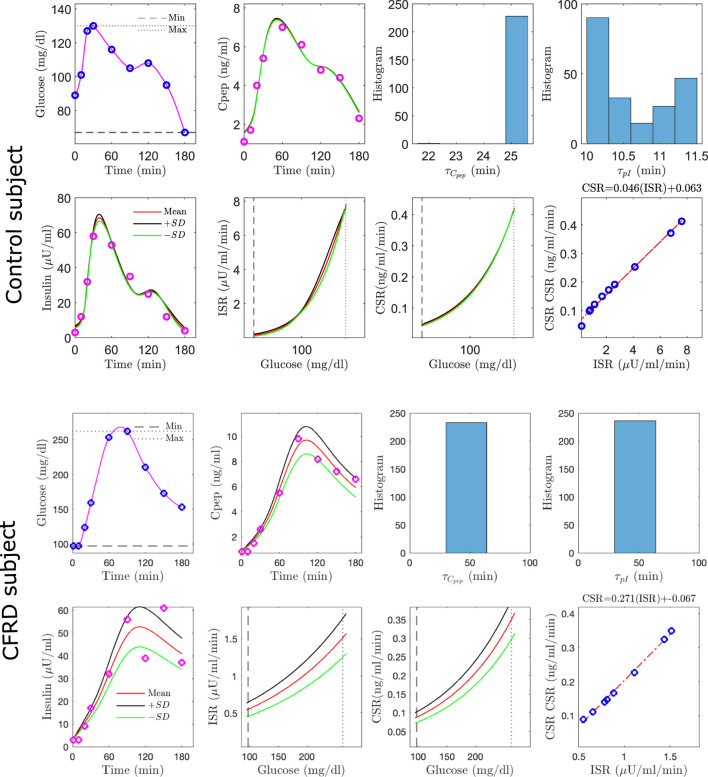
Estimation results, using the algorithm with the single compartment model, for a single control (Top) and a single CFDR (Bottom) subject. Each composite includes **(A)** glucose measurements (blue circles) and the interpolated glucose (magenta line) used as model input **(B)** measured c-peptide (circles) and model-generated mean c-peptide (magenta line) and ±standard deviation (black, green lines); histograms of inferred degradation time for c-peptide **(C)** and insulin **(D)**; **(E)** measured insulin (red circles) and estimated insulin trajectory (magenta with ±SD black, green); mean inferred ISR **(F)** and CSR **(G)** (red with SD black, green); and **(H)** CSR/ISR relationship.

Shown in Figure 4 are stereotypical results for control (upper group) and CFRD subjects (lower panels). The actual glucose measurements (blue circles) and interpolated glucose (magenta), used as an input to the algorithm, are shown in the Figure 4A. Also, the measured c-peptide (red circles) and estimated c-peptide (magenta with standard deviation (±SD) black, green) are presented in the Figure 4B. Histograms of the inferred degradation times are shown for both c-peptide (Figure 4C) and insulin (Figure 4D). Measured insulin (red circles) and estimated insulin (magenta) are shown (with ±SD black, green) in the Figure 4E. Estimated ISR and CSR are presented with ±SD (black, green) are shown in the Figures 4F,G for the time points at which data was taken.

In both examples, the relationship between CSR and ISR closely matches a linear fit, with slope of order the expected value of 0.056 = ng/*μ*U.

### 3.3.1 Quantification of Goodness of Fit

We quantify the goodness of fit of three different features of these fits the measured values; how well the trajectory of the modeled insulin (*I* (*t*|**Θ**)) fits the measured values, how well the trajectory of the modeled c-peptide (*C* (*t*|**Θ**) fits the measured c-peptide values; and goodness of the linear fit between the CSR and ISR, $\tilde{CSR}\left(ISR\right)$ . In each of these cases, we use normalized root-mean-square (RMS) errors:

$$RMS_{I_{k}}=\frac{1}{\bar{I_{k}}}\sqrt{\underset{k}{\sum }\left(\right.I_{k}-I{\left(t_{k}|\hat{\Theta }\right)}^{2}}$$ (10a)

$$RMS_{C_{k}}=\frac{1}{C_{k}¯}\sqrt{\underset{k}{\sum }\left(\right.C_{k}-C{\left(t_{k}|\hat{\Theta }\right)}^{2}}$$ (10b)

$$RMS_{CSR\left(ISR\right)}=\frac{1}{CSR_{k,max}}\sqrt{\underset{k}{\sum }\left(\right.CSR\left(t_{k}\right)-\tilde{CSR}{\left(ISR\left(t_{k}\right)\right)}^{2}}$$ (10c)

where ${\bar{I}}_{k}$ is the mean of the measured insulin values, ${\bar{C}}_{k}$ is the mean of the measured c-peptide values, and *CSR*_*k*, max_ is the maximum CSR value.

**FIGURE 5:**
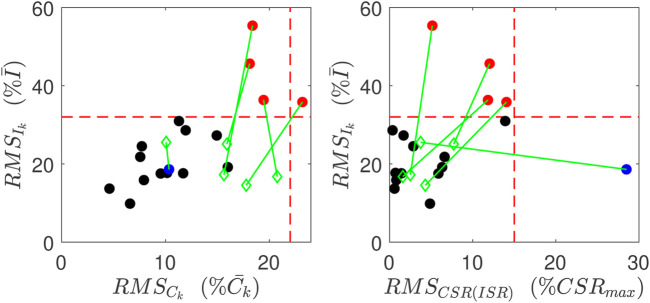
Goodness of Fits for normal subjects. Red points indicate have relatively poor reconstruction of insulin measurements, and the blue point has relatively poor linear relation between CSR and ISR. These metrics for these poor reconstructions are all improved (green points) when the data point at 60 min is left out.

Estimation results are obtained and evaluated for all control and CFDR subjects. Based on the goodness of fit values (Eq. 10), our algorithm achieved relatively good estimates of ISR and CSR for 12 of 17 (71%) control subjects and 7 of 9 (78%) CFRD subjects. Error estimates are shown in Figure 5 plotted for the output for each of the control subjects (filled circles). As can be seen in the left panel, four subjects had very high RMS error in reconstruction of both insulin trajectories (red). In addition, at least one subject’s fit had especially poor linear relationship between CSR and ISR (blue).

### 3.3.2 Identification of Potential Outlier Data Points

We hypothesize that for these data sets, approximately 30% of the subjects’ data have at least one outlier data point that is sufficient to corrupt the inference. Such outliers would also interfere with clinical diagnostics, and therefore the ability to identify and correct for these outliers would be a substantial gain.

**FIGURE 6:**
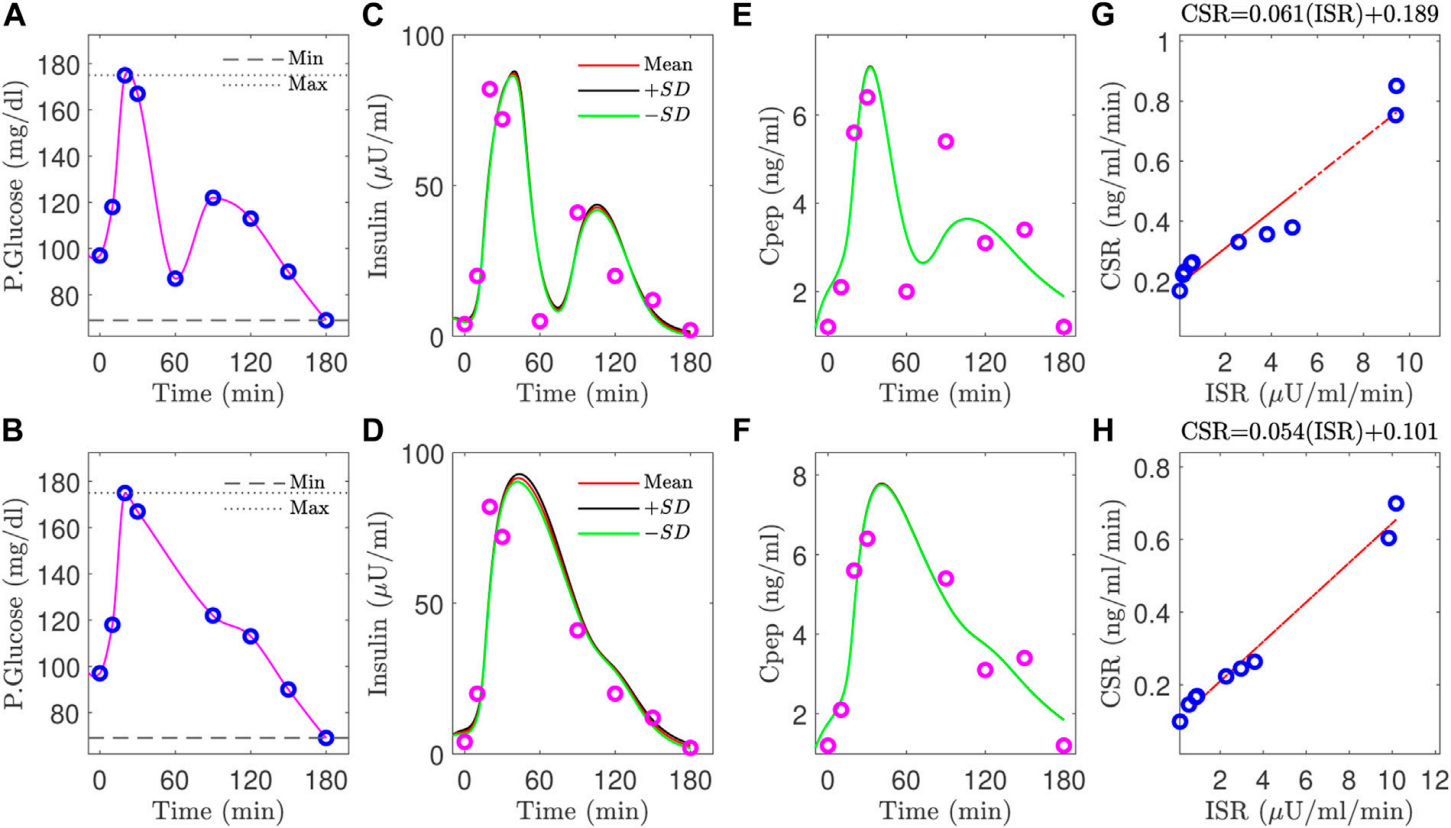
Improvement for the estimation results of plasma insulin (**(D)** versus **(C)**), c-peptide (**(F)** versus **(E)**), and slope (**(H)** versus **(G)**) by removing the uncertain blood glucose value at 60 min **(A)**, and using the interpolated glucose values in the gap between the glucose values at 30, 90 min **(B)**.

In the five poorly estimated control subjects, we observed glucose values that had rather severe dip at 60 min, and then a recovery to a middle value, as illustrated in Figure 6A, which we suspect may be in error.

To test our interpretation for poor estimates, we consider one control subject with an uncertain glucose measurement that dropped at 60 min, from 140 mg/dl to 85 mg/dl. We then removed this 60 min glucose value and the associated insulin and c-peptide measurements. Since we use glucose interpolation as an input to the model, the gap between the glucose values between 30 and 90 min is filled by the interpolated glucose values, as shown in Figures 6A,B. Without the value at 60min, the insulin and c-peptide measurements are used to re-estimate the parameters. As shown in (D, F), the computed insulin and c-peptide trajectories better match the residual measured values, and the relationship between CSR and ISR is better fit by a line (H).

Quantitatively, all three of the RMS errors improved for this subject, as did the errors for all five subjects whose fits were previously identified as having high error. The improvement is illustrated by the green diamonds in Figure 5. The green lines link the improved error values with the error values prior to this analysis.

In contrast, for all other subjects, if the same 60 min time point was left out the errors did not significantly degrade.

Note that the objective function (Eq. 9) that is minimized with the optimization is only sensitive to the model reconstructed values at the measured times. The model dynamics (insulin or c-peptide accumulation) are substantially only sensitive to the ISR or CSR within approximately one degradation time constant (*τ*_*x*_), which for controls is of order 15 min. Therefore the optimization is primarily sensitive to the interpolated glucose trajectory $\left(\bar{g}\right)$ over approximately *τ*_*x*_ ahead of measured data points. This means that as long as the measurements sufficiently sample the glucose dynamics, this method should be robust to dropping out individual data points.

This suggests that the poor estimate comes from the data, not the method.

In contrast, for in the two poorly estimated CFRD subjects, the glucose value dramatically increased to greater than 250 mg/dl within 40 min. This sharp increase in glucose level reduces the amount of time at intermediate glucose levels. As a consequence, it makes estimating ISR difficult at those intermediate values.

### 3.3.3 Inferred Parameters

In Table 1, we provide a summary for the 12 normal and 7 CFRD subjects who were estimated well, including the ISR average values of the estimated parameters presented by the mean and 95% confident interval, slope, and the ISR evaluated at the glucose value of 140 mg/dl. Note that only 9 of 13 control subjects have peak glucose values that reached 140 mg/dl, whereas all of the 7 CFRD had blood glucose of 140 mg/dl or greater.

**TABLE 1:** A summary for the normal and CFRD subjects, including the ISR average values of the estimated parameters, presented by the mean and 95% confident interval, slope, and the ISR evaluated at the glucose value of 140 mg/dl.

Parameter	*τ*_*PI*_	*K*_*m*_	*C*_0_	*α*	Slope	ISR(140 mg/dl)
Control Subject	15 ± 5	123 ± 57	209 ± 39	0.06 ± 0.02	0.06 ± 0.01	128 ± 57
CFDR Subject	28 ± 12	75 ± 60	600 ± 240	0.014 ± 0.01	0.14 ± 0.06	73 ± 60

As a final external validation of the method, we were able to differentiate CFRD from normal patients in two ways. First, as shown in Table 1, the ISR at a glucose of 140 mg/dl is higher for the control subjects than the CFRD subjects. This result indicates the ability of the beta-cells for healthy subjects to produce more insulin to mitigate the increased glucose level. Second, the estimated *τ*_*pI*_ for CFRD subjects is larger than the control subjects, as shown in Table 1. This result reflects physiological insights for CFRD that insulin takes a longer time to accumulate and reach a high value than in the control subjects, due to Lower production in beta cells and lower peripheral degradation rate. Therefore, the increased glucose values in CFRD subjects provide better dynamics for estimation that allows the algorithm to estimate *τ*_*pI*_ more precisely.

The peak of the ISR can be estimated if the glucose range is wide, e.g., 100–350 mg/dl. However, in a short glucose range of 100–140 mg/dl, accurate estimation of the peak is not guaranteed. This observation means it is more likely for CFRD subjects to capture the ISR peak than the control subjects due to the high glucose range in these individuals. We found that two CFRD subjects from among the CFRD group had a glucose range that allowed us to estimate the ISR peak and hence the full ISR functional shape. In these two CFRD subjects, the blood glucose range is between 100–400 mg/dl. On the other hand, the blood glucose range for control subjects is between 95–140 mg/dl, which makes estimating the ISR peak hard to achieve. However, we found only one control subject that the ISR peak was nearly estimated. The glucose range in this control subject is between 95–180 mg/dl. Therefore, we conclude that the peak of the ISR can be better estimated for CFRD subjects, in which the range of blood glucose is wide.

**FIGURE 7:**
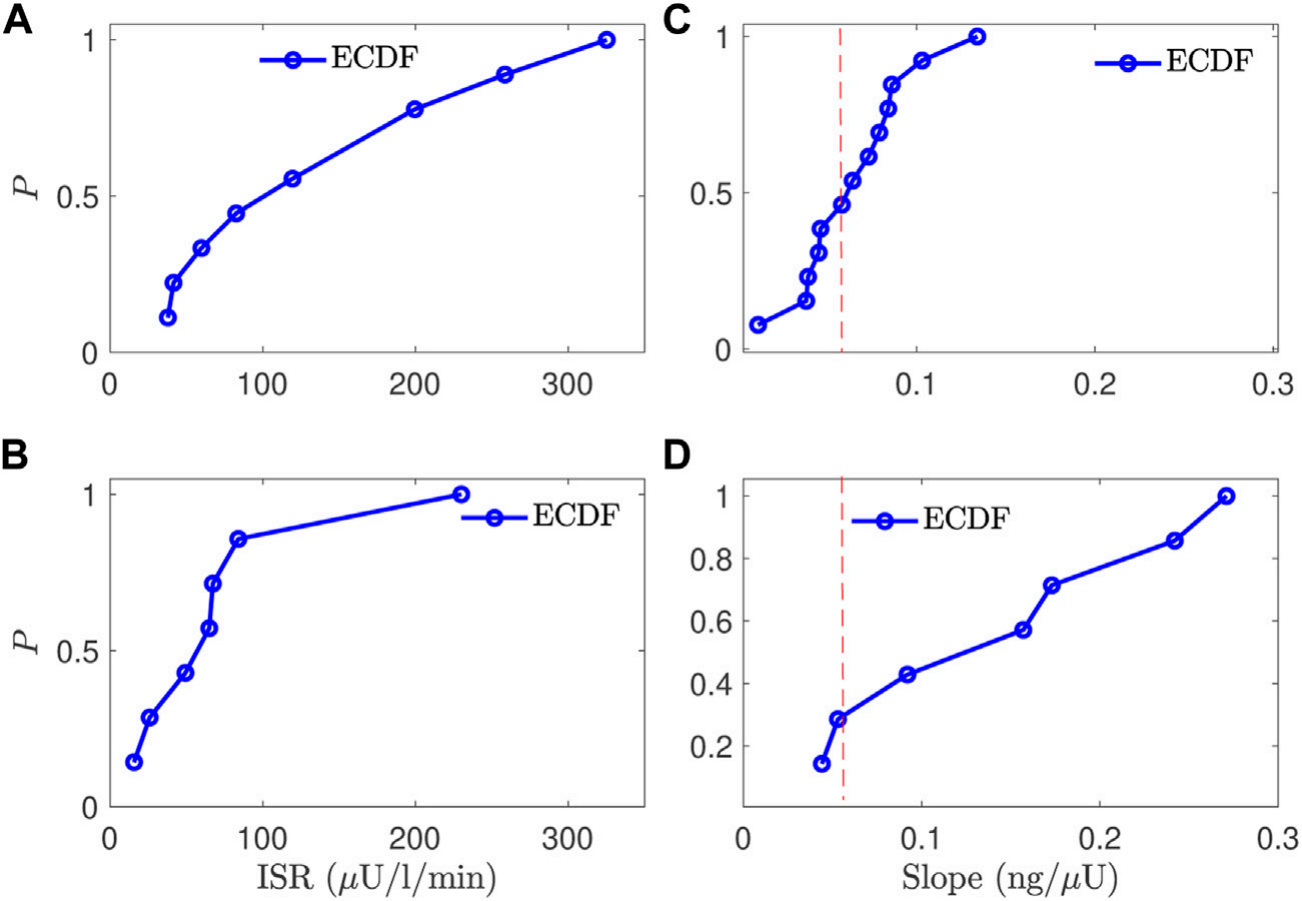
A comparison between the normal and CFRD subjects, using the probability of empirical cumulative distribution function (ECDF), for the ISR value **(A,B)** evaluated at the glucose value of 140 mg/dl (horizontal) and the slope between ISR and CSR **(C,D)**, the vertical axis is the probability (ECDF); the expected value is 0.056 for the slope (red line **(C,D)**).

To compare the normal and CFRD subjects, we evaluated the estimated ISR at the glucose value of 140 mg/dl for subjects with good estimation in the two groups. After removing the poorly fitted subjects and the control subjects that had not reached the glucose value of 140 mg/dl, we obtained 12 control subjects and 7 CFRD subjects out of 17 and 9 subjects, respectively. The results are presented using the empirical cumulative distribution function (ECDF), in Figures 7A,B. Therefore, we found that the ISR values of the normal subjects were with 50% that the ISR exceeds the rate of 100 *μ*U/ml/min. Whereas, the CFRD subjects were with 50% that the associated ISR value around 15 *μ*U/ml/min. These results indicate that the pancreatic beta-cell of the CFRD cannot produce enough insulin due to the dysfunction of these beta-cells. On the other hand, these beta-cells can produce more insulin at the value of glucose (140 mg/dl) in normal subjects.

The estimated slope between the estimated ISR and CSR was also used to characterize these two groups. The slope (CSR/ISR) between the estimated CSR and ISR for both normal and CFRD subjects was plotted in Figures 7C,D, as ECDF. In both control and CFRD subjects we observed a straight line describing the physiological relationship between ISR and CSR. The slope is in the unit of ng/*μ*U. When both units are converted to moles, the expected conversion factor is 0.056. In Figures 7C,D, we plotted this value (0.056) as a vertical (dashed-red) line to illustrate how these slopes, which are predictions of the expected value 0.056, are close to this expected value. As shown in Figure 7C, the predicted slope of the control subjects was around the expected value of 0.056. On the other hand, the wide glucose range in the CFRD subjects used to estimate both ISR and CSR, which gives more information to estimate ISR and CSR trajectories (e.g., ISR secretion and peak regions), increased the uncertainty in the estimated slope, as shown in Figure 7D.

### 3.3.4 Two-Compartment Model

Note that the above fits and figures were obtained using the single-compartment model. However, comparable results can be obtained when incorporating the two-compartment model with the algorithm. But, it is a significant to note that, when the single model cannot estimate the patient’s ISR and CSR, adding a second compartment is not helpful. For a comparison between the two models, the ISR was evaluated at the glucose value of 140 mg/dl, and the slope between ISR and CSR was estimated, for control subjects, using our algorithm incorporating the two models. Therefore, shifting to the two-compartment model, the control subjects’ data gives a fraction difference of absolute mean error for the ISR at glucose value of 140 mg/dl provided by (Mean ± SD) 0.32 ± 0.2. In contrast, the fraction difference of the absolute average error of the slope is given by 0.14 ± 0.15. These results indicate that adding more compartments and unknown parameters is unnecessary to estimate reliable ISR. Instead, a simple model can be incorporated with our method to estimate ISR for people with different beta-cell functions.

## 4 Discussion

We developed a new estimation approach for inferring the ISR from plasma insulin and c-peptide measurements. We validated this method with synthetic data and nominal physiological parameters and were able to reconstruct these values from generated ground truth data with 20% noise is added to each data point for both plasma glucose and insulin. Then the algorithm was applied to OGTT clinical data for both control and CFRD subjects. We use the estimated slope between ISR and CSR to evaluate the estimation for both normal and CFRD groups, as well as the RMS between the observations and the model-estimated values.

We hypothesize that to estimate ISR, it is not necessary to use an OGTT, IVGTT, or other glucose tolerance test. Instead, it can be estimated by knowing glucose values and a nonlinear function of the secretion rate with unknown parameters. Therefore, we specifically implemented a sigmoid function to model the ISR and CSR and then estimate them independently from insulin and c-peptide data. The ISR peak can be estimated, using this function, if the glucose value is high enough to capture the peak. Using our method, we expect to estimate the baseline of the secretion rates if we have more sampled data, especially at the beginning of the test.

### 4.1 Validation

Our validation test uses the equal molar ratio between plasma insulin and c-peptide secretion rates. Even though the CFRD and control subjects have different physiology and the ISR and CSR have various physiological parameters and nonlinear relations with plasma glucose concentration, our algorithm recovers the linear relationship between ISR and CSR for both groups. This result indicates the accuracy of the estimation of the algorithm. Another test uses the normalized root-mean-square (RMS) error between the estimation and the measured values. We showed that the estimation, in the two groups, in which the relationship between ISR and CSR is linear, the RMS error between modeled insulin or c-peptide and estimated ones is small. This observation reflects the consistency in our results showed by these two validation tests used in our method.

### 4.2 Phenotype

We were able to differentiate the normal and CFRD diabetes phenotypes. We show that the ISR for individuals with CFRD is statistically significantly lower than the ISR for individuals’ normal glucose regulatory systems (see Table 1). However, the ISR peak for the two groups did not differentiate them because the peak ISR was often not observable or computable for normal patients. In addition, due to the high glucose dynamics and slow insulin accumulation in the CFRD subjects, which reveals more information about the insulin degradation time (*τ*_*pI*_), the estimated *τ*_*pI*_ was larger in this group than the control subjects.

### 4.3 Identifying Potentially Erroneous Data Points and Improving Reliability

As shown in Section 3.3.2, this inference method may be able to identify erroneous data points. In the example shown (Figure 6, and green point in Figure 5), such identification leverages three separate components: the goodness of fit of the insulin trajectory, the goodness of fit of c-peptide trajectory, and the linear relationship between ISR and CSR. Because both ISR and CSR are inferred independently, these three are independent measures.

The inference relies on the physiological knowledge that ISR is primarily a function of plasma glucose concentration, and the linear relation embodies the physiological fact that c-peptide and insulin are released in a 1:1 molecular ratio. Such use of external knowledge - that ISR is primarily a function of plasma glucose, and that CSR is proportional to ISR - is a simple and principled pathway to identifying data errors.

We note also that because the underlying model for plasma insulin or c-peptide accumulation includes a degradation time that appears to be in the range of *τ*_*I*_ 5–15 min, the model is in effect only dependent on the interpolated glucose values within *τ*_*I*_ or *τ*_*C*_ ahead of each data point, and for the distally separated points residual after removal of the point at 60 min, this time is relatively small.

If proven reliable in future studies, we anticipate that such analysis - and removal of erroneous points - could make clinical testing more reliable by increasing the amount of diagnostic information that can be extracted from a single diagnostic test, and decrease the need for multiple diagnostic tests using model-based inference. Currently, the ADA recommends four pathways for diagnosing pre-diabetes and type-2 diabetes, one of which includes an OGTT (Davidson et al., 2021b). Similarly, the recommendation to diagnose gestational diabetes is via a GTT or OGTT (Davidson et al., 2021a). In both cases, a diagnosis requires two tests. By using inference paired with the information in the dynamics of the OGTT rather than a single value, we suspect it would be possible, as we show in this work (Figure 6), to remove inaccurate outliers and accurately estimate ISR and other diagnostic quantities. If corroborated with further studies, this should motivate quantification of *both insulin and c-peptide* from blood draws during such clinical measures.

### 4.4 Inferring Pancreatic Health

Additionally, the model provides a platform for extracting additional information. For example, here we estimate the entire ISR curve, increasing accuracy and explainability of the context of the patient state, leading to quantified information regarding how much of the ISR was observed for observed glucose levels and how much excess capacity for insulin production the patient may have, leading to more accurate diagnosis of the patient’s endocrine state.

Considering the above results and discussion, we now have a suitable method with physiological insights about estimating ISR for subjects with different physiological conditions. Furthermore, we showed that using a simple model is good enough to estimate ISR rather than a more complex model with more compartments and unknown parameters. Moreover, we found that using the two compartment, when the single compartment failed to estimate the ISR and CSR correctly, is not useful. These results allow us to implement the estimated ISR function into glucose models with various fidelity and complicity to understand better the glucose regulation system for patients with different pancreatic beta-cell functions.

### 4.5 Hepatic Insulin Degradation

We note that in this modeling we have lumped all insulin and c-peptide degradation to a general degradation rate, and have not tried to differentiate hepatic degradation or its effects. In the ISR literature (i.e., Watanabe et al., 1998; Watanabe and Bergman, 2000), such efforts are motivated because the pancratic beta-cells secrete insulin and c-peptide into the portal vein blood stream. The portal vein then passes through the liver and some insulin (up to 80%) is immediately degraded by the hepatocrytes (Najjar and Perdomo, 2019). If this process were simply proportional to plasma insulin concentration, then the one-compartment model for insulin would be modified to:

$${\dot{x}}_{pi}=u_{i,p}\left(t\right)\left(1-\alpha_{h}\right)-\left(\kappa_{i}+\beta_{port}\alpha_{h}\right)x_{pi}$$ (11)

Here *u*_*i*,*p*_ is the ISR at the pancrease into the portal vein, *α*_*h*_ is the absorption proportionality *α*_*h*_ ∈ (0 : 1), and *β*_*port*_ is the ratio of the portal blood flow rate to the total blood volume, which for adult humans *β*_*port*_ ∈ (0.15–0.4)/s. Likewise, *κ*_*i*_ is the degradation proportionality due to other processes. The addition to the standard degradation rate comes because the liver cannot distinguish between freshly secreted insulin and circulating insulin.

For the work as described, the ISR inferred is effectively the rate of insulin secretion into the circulation system following transit through the liver, i.e., *u*_*i*,*inferred*_ = *u*_*i*,*p*_(*t*) (1 − *α*_*h*_). Because c-peptide is not primarily degraded in the liver, this correction factor doesn’t apply. Therefore in cases where both insulin and c-peptide are measured, the slope of the linear relation between parametrized CSR and ISR should be equal to 1/(1 − *α*_*h*_) (after unit conversion to molar units). For normal subjects, the majority of subjects therefore had hepatic absorption ratios of *α*_*h*_ < 0.4 (Figure 7C). But the CFRD subjects had wider range of slopes, consistent with values as high as *α*_*h*_ < 0.8.

Because our inference method also estimates the insulin degradation rate for an individual, this model implies that *β*_*port*_ *α*_*h*_ < 1/*τ*_*pI*_. The inferred fit for normal subjects, with degradation times are of order *τ*_*pI*_ are of order 10−15 min and *α*_*h*_ 0.1, are consistent with this inequality and reasonable values of *β*_*port*_. But, for example, the CFRD subject whose data is shown in Figure 4, has an *α*_*h*_ 0.8 and *τ*_*pI*_ ∼50 min, which is not consistent with normal portal blood flow values.

In future work with larger data sets we will investigate these observations as a function of subject health.

We further note that this linear model (11) for hepatic insulin degradation has limitations. In particular, the insulin-receptors on the hepatocytes then signal insulin endocytosis and degradation (Najjar and Perdomo, 2019). Therefore the rate of degradation is insulin dependent.

### 4.6 Study Limitations

This work represents a first attempt to apply this modeling approach to infer the parametrization of the ISR from clinical data. Here we have applied this approach to rather small data sets for both control and CFRD subjects. We anticipate that the distribution of normal and abnormal ISR functions will only be clear from much larger sets. We note that this effort fall short in terms of the aim of establishing functional shape for control subjects. This method can only infer the ISR function over the range expressed during the clinical measurement, and the maximum glucose level for control subjects represented here was well below 150mg/ld - and the inferred ISRs were far from saturated.

## 5 Conclusion

This study presents a new approach for estimating ISR using plasma insulin and c-peptide measurements. Our approach uses simple insulin and c-peptide models and applies both insulin and c-peptide measures. This algorithm can infer ISR and CSR from OGTT data. Additionally, the method provides a deeper interpretation of the OGTT and a measure of the robustness and accuracy in both the inference and data.

We validate the estimation results in three ways. First, we validate the results by estimating the plasma insulin and c-peptide and comparing RMS between measurements and modeled responses of these variables. Second, we use the 1:1 M ratio between ISR and CSR to assess the estimation results. We showed that a linear relationship between ISR and CSR can be observed when they are estimated correctly. This result is confirmed in both CFRD and normal subjects. Third, we showed that our algorithm can differentiate between subjects with different beta-cell phenotype-related diseases. Moreover, we showed that the ISR level in CFDR subjects is lower than the ISR level in normal subjects. However, since the variation in blood glucose is high in CFRD patients, the peak of ISR and plasma degradation time of insulin and c-peptide are estimated more precisely. Further, we showed that the estimation of ISR utilizing the single-compartment model is very similar to the results using the two-compartment model. This indicates different models the robustness of our approach in estimating the ISR using different models’ complicity and confirms that the ISR can be estimated precisely using only a simple model with less parameters. We also tested our model by treating uncertain measured values in the data. Finally, we provided a physiological interpretation that our method can handle the uncertainty in measured values and improve the estimation of ISR.

The immediate impact of this work is the development of a new approach for estimating ISR, which is now available for determining the beta-cell secretion rates for people with different conditions. This method is ready to implement into glucose models providing a better understanding of the glucose regulation system and monitoring people with diabetes.

## Code Availability

Code for both fitting the ISRs, CSRs, and parametrizing the relationship between CSR and ISR, with examples to model-generated data, are available at (Inferring Insulin Secretion Rate From Sparse Patient Glucose and Insulin Measures (hyperlink = https://scholarsphere.psu.edu/resources/20d599c4-93fb-4a62-81d8-b69a88d58627) at doi: 10.26207/e8rf-e082).

## Data Availability

The datasets presented in this article are not readily available because The request should be submitted to the Colorado Multiple Institutional Review Board (Aurora, CO). Requests to access the datasets should be directed to Name: CC, email: ChristineL.Chan@childrenscolorado.org.
